# Relationship between the COMT-Val158Met and BDNF-Val66Met Polymorphisms, Childhood Trauma and Psychotic Experiences in an Adolescent General Population Sample

**DOI:** 10.1371/journal.pone.0079741

**Published:** 2013-11-05

**Authors:** Hugh Ramsay, Ian Kelleher, Padraig Flannery, Mary C. Clarke, Fionnuala Lynch, Michelle Harley, Dearbhla Connor, Carol Fitzpatrick, Derek W. Morris, Mary Cannon

**Affiliations:** 1 Department of Psychiatry, Royal College of Surgeons in Ireland, Beaumont Hospital, Dublin, Ireland; 2 National Centre for Suicide Research and Prevention of Mental Ill-Health, Karolinska Institutet, Stockholm, Sweden; 3 Lucena Clinic, Tallaght, Dublin, Ireland; 4 Department of Child and Adolescent Psychiatry, St Vincent’s Hospital, Fairview, Dublin, Ireland; 5 School of Medicine & Medical Science, University College Dublin, Dublin, Ireland; 6 Neuropsychiatric Genetics Research Group, Department of Psychiatry and Institute of Molecular Medicine, Trinity College Dublin, Dublin, Ireland; Consiglio Nazionale delle Ricerche (CNR), Italy

## Abstract

**Objective:**

Psychotic experiences occur at a much greater prevalence in the population than psychotic disorders. There has been little research to date, however, on genetic risk for this extended psychosis phenotype. We examined whether COMT or BDNF genotypes were associated with psychotic experiences or interacted with childhood trauma in predicting psychotic experiences.

**Method:**

Psychiatric interviews and genotyping for COMT-Val158Met and BDNF-Val66Met were carried out on two population-based samples of 237 individuals aged 11-15 years. Logistic regression was used to examine for main effects by genotype and childhood trauma, controlling for important covariates. This was then compared to a model with a term for interaction between genotype and childhood trauma. Where a possible interaction was detected, this was further explored in stratified analyses.

**Results:**

While childhood trauma showed a borderline association with psychotic experiences, COMT-Val158Met and BDNF-Val66Met genotypes were not directly associated with psychotic experiences in the population. Testing for gene x environment interaction was borderline significant in the case of COMT-Val158Met with individuals with the COMT-Val158Met Val-Val genotype, who had been exposed to childhood trauma borderline significantly more likely to report psychotic experiences than those with Val-Met or Met-Met genotypes. There was no similar interaction by BDNF-Val66Met genotype.

**Conclusion:**

The COMT-Val158Met Val-Val genotype may be a genetic moderator of risk for psychotic experiences in individuals exposed to childhood traumatic experiences.

## Introduction

Much research has established that psychotic experiences have a higher prevalence in the general population than psychotic disorders [1-4]. A meta-analysis of all community studies of psychotic experiences in children and adolescents found a median population prevalence of 17% in children aged 9-12 years and 7.5% in those aged 13-18 years [5]. Psychotic experiences in adolescence are associated with high risk for severe psychopathology, both in the immediate term and later into adulthood, including both psychotic [6-8] and non-psychotic disorders [9-12]. Psychotic experiences share many important risk factors with schizophrenia [13]. For example, in the case of familial risk [14], there is covariation of psychotic experiences with maternal schizophrenia-spectrum disorder [15]. In common with psychotic disorders, psychotic experiences are more prevalent in adolescents who have had traumatic experiences, including physical abuse, exposure to domestic violence and unwanted sexual experiences [16-27]. 

We previously suggested that genes for psychosis may, in fact, be genes for the broader ‘extended psychosis phenotype’, made up not just of individuals with psychotic disorders but also a much larger population of individuals with psychotic experiences [28]. Investigation of the genetic aetiology of the extended psychosis phenotype may provide novel insights into the genetic underpinnings of psychosis. 

Two frequently studied genetic variants in psychosis research are the catechol-o-methyl-transferase (COMT) Val158Met and the brain-derived neurotrophic factor (BDNF) Val66Met polymorphisms [29-32]. COMT plays an important role in the metabolism of catecholamines, such as dopamine and norepinephrine in the central nervous system. The Val158Met polymorphism is associated with a 3- to 4-fold variation in enzymatic activity [33,34] between the high activity Val/Val genotype and the low activity Met/Met genotype. Diverse gene-environment interactions have been reported in the case of COMT-Val158-Met, particularly in moderating risk for psychotic disorder [35,36], for example, in the case of cannabis use in adolescence [36,37]. Indeed, recent research has identified gene-gene-environment interaction involving COMT in the case of subjectively reported daily life stress using an experience sampling method in patients with schizophrenia [38]. Furthermore, the COMT-Val158Met Val allele has been associated with self-reported psychotic experiences in the context of stress and cannabis use in a Dutch adult population sample [39]and with the stress of army induction in a Greek male conscript sample [40]. COMT-Val158Met has also been associated with increased schizotypal personality trait scores in Val-Val individuals exposed to higher levels of self-reported childhood trauma [41]. BDNF, on the other hand, has an established role in neuronal development and cell survival in response to stress [42] and is abnormally expressed in schizophrenia [43]. Interestingly, the BDNF-Val66Met polymorphism has been shown to moderate the impact of childhood adversity on later expression of affective symptoms [44,45], suggesting the possibility of gene-environment interactions and one study reported an interaction between childhood abuse and Val66Met in predicting psychotic experiences [29]. 

### Aims of the Study

The present study aimed to explore the role of the COMT-Val158Met and BDNF-Val66Met polymorphisms in two community samples with psychotic experiences. We wished to test for a direct association between these polymorphisms and psychotic experiences in the general population. Furthermore, we aimed to test a gene-environment interaction, specifically, that those with the COMT-Val158Met Val-Val genotype or the BDNF-Val66Met Met-Met/Val-Met genotypes are more susceptible to psychotic experiences following trauma than those with other genotypes.

## Materials and Methods

### Sample

**Figure 1:**
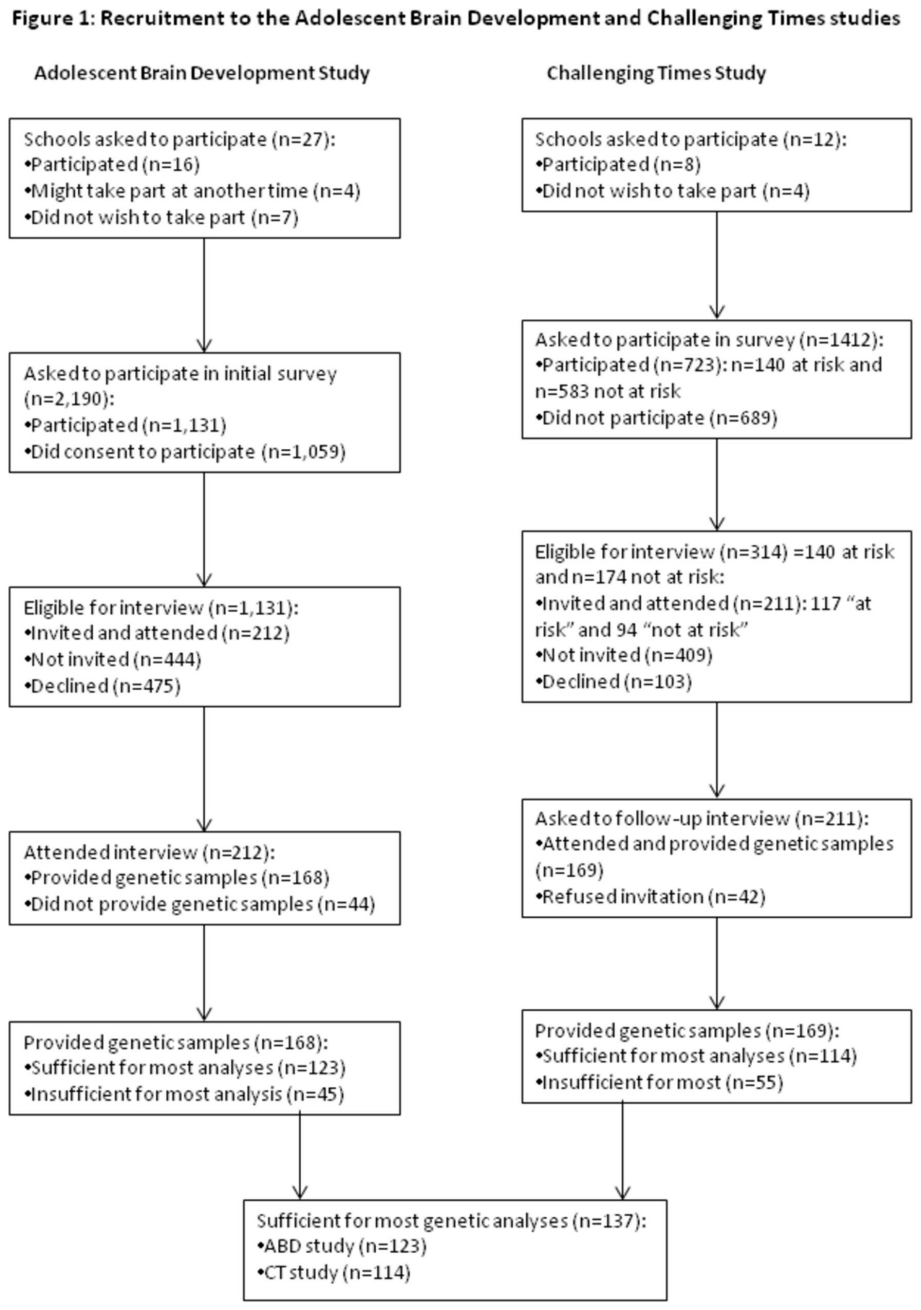
Recruitment to Adolescent Brain Development and Challenging Times studies.

The sample consisted of 237 adolescents from two independent studies: 123 participants from the Adolescent Brain Development (ABD) study and 114 participants from the Challenging Times (CT) study. The study methodologies have been previously reported [46-48]. Details of recruitment for both studies are presented in Figure 1.

Briefly, the ABD study took place in counties Dublin and Kildare, Ireland, which contains a mixture of urban and suburban housing types of different socioeconomic status. Out of twenty seven schools approached, sixteen agreed to participate (59.3%), while four said they may participate at another time and seven (25.9%) declined participation. Participants were aged 11-13 years and were in the two most senior classes in the Irish primary school system. Written parental consent was required for adolescents to take part and 1,131 signed consent forms were returned out of 2,190 distributed (52%). Of the 1,131 adolescents who took part in the survey study, 656 (58%) indicated an interest in participating in the interview study and a random sample of 212 of these attended for interview. Those who participated at interview did not differ from those who did not on levels of psychopathology as determined by the SDQ. Those who attended for interview were also representative of the Irish population in terms of socioeconomic status and ethnic background. Among this sample, a total of 168 individuals provided genetic samples. There was insufficient DNA for analysis in 45 of these samples, resulting in sufficient DNA for genotyping the BDNF-Val66Met or COMT-Val158Met polymorphisms in 123 participants. 

The CT study took place in the geographical catchment area of a child and adolescent mental health team in north Dublin, containing a population of 137,000. This urban area includes pockets of severe inner city deprivation, large suburban housing estates and more affluent areas of private housing. The population includes a higher proportion from lower socioeconomic backgrounds compared to the overall Irish population. A sample of 12 secondary schools in the catchment area was selected using stratified random sampling according to the approximate socio-economic status of the school. Of these 12 schools, eight participated in the study. One school did not participate owing to concerns that questioning pupils about suicidal thoughts might be harmful and three schools that agreed to participate were excluded because insufficient parental consent forms were received (<50%). The exclusion of these schools did not skew the socio-economic distribution of the remaining schools. A total of 743 students aged 12-15 years in eight schools were screened for psychopathology using the Strengths and Difficulties Questionnaire [49], which measures child psychopathology risk, and the Children’s Depression Inventory [50][51], which measures the cognitive, affective and behavioural signs of depression. Written informed consent was obtained from the parent or guardian of participants. One hundred and forty pupils scored above the threshold on these instruments and all of these adolescents were invited to full psychiatric interview, of whom 117 (83.6%) agreed to attend. A comparison group of 173 healthy adolescents, matched for gender and school, were also invited to interview and 94 (54%) agreed to attend. Of these 211, 169 (80%) attended for further follow-up [52] and provided genetic samples. Of these, 55 did not contain sufficient DNA for analysis, resulting in genotyping of 114 participants for the COMT Val158Met or BDNF-Val66Met polymorphisms. 

In total, between both studies, there was sufficient DNA from 226 participants for analysis of COMT-Val158Met (115 from ABD study and 111 from CT study) and 222 participants for analysis of BDNF-Val66Met (116 from ABD study and 106 from CT study). This data is not available online owing to the necessity to protect the confidentiality of the young people who participated (and discussed the sensitive areas of psychotic experiences and history of abuse). However, we are happy to facilitate requests from researchers to freely review the data.

### Genotyping

Genetic analysis was carried out using DNA extracted from saliva samples.  The COMT (rs4680) and BDNF (rs6265) SNPs were genotyped using Taqman® SNP genotyping assays on a 7900HT sequence detection system (Applied Biosystems).  The call rate for the Taqman genotyping was >95% and the samples were in Hardy-Weinberg equilibrium (p>0.05).  Along with these samples, thirteen HapMap CEU DNA samples (www.hapmap.org) were genotyped for each SNP (rs4680 and rs6265) for quality control purposes and were found to be 100% concordant with available HapMap data for these SNPs.

### Exposure and outcome measures

Participants and their parents were interviewed using the Schedule for Affective Disorders and Schizophrenia for School-aged Children (K-SADS), Present and Lifetime versions. The K-SADS is a well-validated semi-structured research diagnostic interview for the assessment of Axis I psychiatric disorders in children and adolescents [53]. Children and parents answered the same questions but were interviewed separately. All interviewers were trained psychiatrists or psychologists. The psychosis section of the K-SADS was used to assess the participants’ psychotic experiences. They recorded extensive notes of potential psychotic phenomena in this section of the interview and a clinical consensus meeting, which took place following the interviews, classified these as a binary variable, having psychotic experiences or not, masked to diagnoses and all other information on the participants. This measure of psychotic experiences was our outcome variable. As part of the interview, a range of traumatic experiences were enquired about from both parent and child, including instances of physical abuse, sexual abuse and exposure to domestic (inter-parental) violence. A report from either child or parent of physical or sexual abuse or of witnessing domestic violence was classified as a childhood trauma [17]. Polymorphism genotypes were converted for ease of analysis into binary variables based on the findings of previous studies. The COMT-Val158Met polymorphism was categorised as either Val-Val or Met-Met/Val-Met, based on research supporting a gene-environment association in the case of Val-Val with transient psychotic experiences and schizotypal traits [40,41]. The BDNF-Val66Met polymorphism was categorised as either Val-Val or Met-Met/Val-Met, based on the previous finding of a gene environment association with psychotic experiences in Met carriers [29].

### Ethics Statement

Ethical approval for the Adolescent Brain Development study was received from Beaumont Hospital medical ethics committee and for the Challenging Times study from the Mater Misericordiae University Hospital medical ethics committee. Informed assent was obtained from participants under age 16 years with informed consent received from their parents. In addition, all individual data was anonymised with no identifying characteristics present. Written informed consent was obtained from the parent or guardian of participants.

### Statistical analysis

Differences between those who participated in interview and provided genetic samples and those who participated but did not were assessed using chi-squared tests. Similarly, differences between those with versus without psychotic experiences were assessed using chi-squared tests or Fisher’s exact test as appropriate. 

Based on the hypothesis that COMT-Val158Met genotype can moderate the influence of childhood trauma on psychotic experiences, we tested whether psychotic experiences would be predicted by an interaction between a gene (either COMT-Val158Met or BDNF-Val66Met) and an environment (childhood trauma). To analyse the interaction between childhood trauma and genotypes (COMT-Val158Met and BDNF-Val66Met), logistic regression was used, with the absence or presence of psychotic experiences as the dependent variable. Sex, school grade and cannabis use were used as covariates. School grade was included as a categorical variable with four levels, one for each grade. The first two grades were recruited for the ABD study and the second two grades for the CT study. This variable therefore approximated the effects of both age and study group. Cannabis use and sex were also included to control for potential confounding by these factors. A logistic regression model was first fitted to determine the main effects of childhood trauma and COMT-Val158Met genotype (model 1). Second, to explore the interaction effect of COMT-Val158Met and childhood trauma, a second model was fitted with an interaction term (model 2) and this was compared to the first model using the likelihood ratio test. This process was also repeated separately in the same manner to examine the main effect of BDNF-Val66Met genotype and interaction between this genotype and childhood trauma. 

Finally, where these tests indicated an important G X E interaction, the effect of this interaction was explored within each genotype group, using stratified tables with odds ratios and 95% confidence intervals. Statistical analyses were conducted using Stata version 11.0 for Windows. All statistical tests were two-sided; P-values less than 0.05 were judged statistically significant.

## Results

### Descriptive statistics

Those who attended for interview and provided usable genetic samples were broadly similar to those who attended for clinical interview but did not provide usable genetic samples (details in table 1), though a higher proportion of males and a lower proportion of those in the highest school grade provided usable genetic samples.

**Table 1:** Comparison of those with genetic data to those without genetic data, according to demographic factors, trauma and psychotic experiences.

		Genetic data				
Variable		No (n=208) (%)	Yes (n=237) (%)	χ^2^	Df	P-value~
Sex	Male	78 (38)	128 (62)	11.25	1	0.001*
	Female	127 (54)	109 (46)			
School grade	5^th^ grade	41 (43)	55 (57)	10.83	3	0.013*
	6^th^ grade	64 (48)	68 (52)			
	7^th^ grade	47 (37)	79 (63)			
	8^th^ grade	49 (60)	33 (40)			
Social class	Class I/II	77 (39)	118 (61)	2.61	2	0.272
	Class IIIN	50 (38)	81 (62)			
	Class IIIN/IV/V	12 (27)	33 (73)			
Psychotic experiences	No	177 (47)	200 (53)	0.11	1	0.742
	Yes	30 (45)	37 (55)			
Traumatic experiences	No	193 (48)	216 (53)	1.01	1	0.315
	Yes	13 (37)	21 (62)			
Cannabis use	No	131 (37)	226 (63)	1.95	1	0.162
	Yes	10 (53)	9 (47)			

Df=degrees of freedom; ~P-values based on Chi-squared test or Fisher’s exact test as appropriate

The sample was 54% male and all participants were of white European origin. Psychotic experiences were present in 37/237 (17%) of the total sample. Childhood trauma was reported by 21/237 (9%). This included 13 (5.5%) who witnessed domestic violence, 9 (3.8%) who described child physical abuse and 4 (1.7%) who described child sexual abuse. There was overlap in these individuals, with some experiencing multiple forms of childhood trauma. Table 2 presents the group characteristics of those with psychotic experiences and those without psychotic experiences. The prevalence of psychotic experiences did not differ significantly according to sex, childhood trauma, cannabis use, COMT-Val158Met genotype or BDNF-Val66Met genotype. Consistent with previous research [5], psychotic experiences were less common in the older age groups, who were assessed as part of the CT study (age 12-13 years P<0.01; age 13-14 years P=0.03).

**Table 2:** Comparison of cases (with psychotic symptoms) and controls, according to demographic factors, trauma and COMT and BDNF genotype.

		Psychosis				
Risk factor (total number)		No (n=200) (%)	Yes (n=37) (%)	χ^2^	Df	P-value~
Sex (n=237)	Male	105 (82)	23 (18)	-	-	-
	Female	95 (87)	14 (13)	1.17	1	0.28
Age (n=235)	10-11 years	39 (71)	16 (29)	-	-	-
	11-12 years	53 (78)	15 (22)	0.79	1	0.37
	12-13 years	76 (96)	3 (4)	16.92	1	0.00
	13-14 years	30 (91)	3 (9)	4.82	1	0.03
Exposed to trauma (n=237)	No	184 (85)	32 (15)	-	-	-
	Yes	16 (76)	5 (24)	1.18	1	0.28
COMT genotype (n=226)	Met-Met/Val-Met	134 (83)	27 (17)	-	-	-
	Val-Val	55 (85)	10 (15)	0.06	1	0.80
BDNF genotype (n=222)	Val-Val	130 (86)	22 (14)	-	-	-
	Met-Met/Val-Met	59 (84)	11 (16)	0.06	1	0.81
Cannabis use (n=235)	No	191 (85)	35 (15)	-	-	-
	Yes	7 (78)	1 (22)	0.30	1	0.59

Df=degrees of freedom; ~P-value is calculated based on Fisher’s exact test.

### Gene-environment interaction between COMT-Val158Met and childhood trauma with respect to psychotic experiences

Table 3 presents the association between childhood trauma and genotype and psychotic experiences, controlling for sex, school grade (which adjusted for study sample and age group) and cannabis use. In the COMT and BDNF models without interaction, childhood trauma was borderline associated with psychotic experiences (COMT model: OR=3.17, P=0.086; BDNF model: OR=3.25, P=0.073). Neither COMT-Val158Met genotype nor BDNF-Val66Met genotype were associated with psychotic experiences in the models without interaction. Inclusion of an interaction term for gene (COMT-Val158Met genotype) X environment (childhood trauma) resulted in a model that was borderline superior (P=0.057) to the model without an interaction term, using the likelihood ratio test. The P-value for the interaction term between COMT-Val158Met X abuse was borderline significant (P=0.063), suggesting further stratified analysis would be appropriate. When interaction between BDNF-Val66Met and childhood trauma was examined in the same way, the model with interaction was not superior to the model without interaction on the likelihood ratio test ((χ^2^<0.01, P=0.958). 

**Table 3:** Results from hierarchical binary logistic regression models.

	Models without interaction		Models with interaction	
	OR (95% CI)	P-value	OR (95% CI)	P-value
**COMT models (n=223)**				
Abuse (0=absent, 1=present)	3.17 (0.85, 11.81)	0.874	1.33 (0.23, 7.62)	0.749
COMT Val158Met genotype (0=Val-Met/Met-Met, 1=Val-Val)	1.07 (0.45, 2.53)	0.086	0.79 (0.31, 2.06)	0.636
COMT Val158Met genotype X abuse	-	-	17.16 (0.86, 344.25)	0.063
**BDNF models (n=218)**				
Abuse	3.25 (0.90, 11.78)	0.073	3.18 (0.67, 15.00)	0.145
BDNF Val66Met genotype (0=Val-Val, 1=Met-Met/Val-Met)	0.98 (0.42, 2.31)	0.970	0.98 (0.39, 2.43)	0.958
BDNF Val66Met genotype X abuse	-	-	1.07 (0.08, 14.92)	0.958

All models controlled for sex, school year (categorical) and cannabis use

OR=odds ratios; 95% CI=95% confidence interval

*Statistically significant (p<0.05)

Following this, the effect of childhood trauma on psychotic experiences was examined in each COMT-genotype sub-group (see table 4). Childhood trauma was associated with psychotic experiences in those with the COMT-Val-Val genotype (OR=7.43, 95% CI: 1.12-49.11), but not the COMT-Met-Met/Val-Met genotype (OR=0.81, 95% CI: 0.17-3.88) (see table 2). There was borderline evidence in support of an interaction between genotype and childhood trauma (p=0.06) on the chi-squared test for interaction, which is a conservative measure of interaction.

**Table 4:** Association between childhood trauma and psychotic experiences, stratified by COMT-Val158Met group.

**Gene**	**Variant**	**Frequency of psychotic experiences**		**OR**	**95% CI**	P-value*	**Test for homogeneity**
		**No trauma**	**Trauma**				
COMT-Val158Met (n=226)	Val-Val (n=65)	7/59	3/6	7.43	1.12, 49.11	0.04	0.06
	Met-Met & Val-Met (n=161)	25/147	2/14	0.81	0.17, 3.88	1.00	

OR, Odds ratio; 95% CI, 95% Confidence Interval

* P-value is calculated based on Fisher’s exact test.

## Discussion

The current study provides, to our knowledge, the first evidence of a gene-environment interaction between the COMT-Val158Met genotype and childhood traumatic experiences in terms of risk for clinically assessed psychotic experiences in the population. When stratified by COMT-Val158Met genotype, individuals with the Val-Val genotype who experienced childhood trauma were borderline significantly more likely to report psychotic experiences compared to adolescents with the Val/Met and Met/Met genotypes. 

### COMT-Val158Met polymorphism and psychopathology

Our findings should be viewed in the context of advances in understanding of the genetic underpinnings of the extended psychosis phenotype. Binbay et al examined familial risk for this extended phenotype, which ranges from psychotic experiences without impairment to clinical psychotic disorder. They suggested that there are high prevalence and low prevalence genetic risks, interacting with environmental risks, operating across the extended psychosis phenotype but particularly in those with clinical psychotic disorders [54]. In this context, the borderline interaction observed here between COMT-Val158Met genotype and childhood trauma in association with psychotic experiences, a relatively common outcome in this age group, may reflect the operation of a high prevalence genetic risk factor. However, the young age of our sample and differences in the clinical significance of psychotic experiences among different age groups suggest caution in approaching this interpretation. 

Our results suggesting a possible interaction between the COMT-Val158Met Val-Val genotype and childhood trauma in association with psychotic experiences are consistent with previous findings regarding gene-environment interactions between COMT-Val158Met and stressful/traumatic experiences in association with the extended psychosis phenotype. Consistent with our findings, the Val allele was associated with self-reported psychotic symptoms interacting with cannabis use in an adult Dutch population sample [39] and with transient self-reported psychotic experiences during the stress of army induction in a sample (aged 18-24 years) of Greek male conscripts [40] and Val-Val genotype has been associated with more enduring schizotypal traits in those with the Val-Val genotype (but not other genotypes) in the context of self-reported childhood trauma in an adult sample [41]. It is interesting that we have found similar associations in adolescents, despite the changing clinical significance of psychotic experiences with increasing age [10]. 

It has become increasingly clear that psychotic experiences at this age mark risk for a wide range of psychopathology, not limited to psychotic disorders [1,2,11,55]. Indeed, a majority of young people in the population who report psychotic experiences have at least one non-psychotic Axis-1 psychiatric disorder [10]. Furthermore, these symptoms are associated with high risk for suicidal behaviour [56-58]. In addition to psychosis outcomes, the COMT-Val158Met polymorphism has been associated with a diverse range of psychiatric outcomes, including rapid-cycling bipolar disorder [59], obsessive-compulsive disorder [60,61], attention deficit hyperactivity disorder [62-64] and suicidal behaviour [65]. COMT-Val158Met has also been shown to interact with environmental stress in association with major depression [66] and post-traumatic stress disorder [67] in the presence of the Met allele. These findings suggest that the COMT-Val158Met polymorphism is associated with diverse outcomes in different environmental contexts. Given the common overlap between psychotic experiences and non-psychotic disorders, examination of COMT-Val158Met and childhood trauma interaction with non-psychotic disorders and/or disorder severity in association with psychotic experiences may be fruitful. 

The molecular mechanisms of gene-environment interactions remain obscure. Childhood trauma-dependent DNA-demethylation has been associated with increased stress-dependent gene transcription and long-term dysregulation of the stress hormone system in the case of FK506 binding protein 5, a regulator of the stress hormone system [68]. Childhood trauma has the potential to alter methylation at other sites on the genome. Methylation and functional studies would be necessary to further explore this in the case of COMT and childhood trauma.

### BDNF and psychotic experiences

We found no association between BDNF genotype and psychotic experiences. Furthermore, we found no gene-environment interaction between BDNF genotype and childhood trauma in predicting psychotic experiences. This differs from the findings of Alemany and colleagues [29], who found that BDNF-Val66Met genotype moderated the effect of childhood abuse on the positive dimension of psychotic-like experiences, measured using the Community Assessment of Psychic Experiences (CAPE) in a sample from a college campus. There are a number of possible reasons for this difference in findings. Firstly, our samples differed in terms of age and assessment methodology. Psychotic experiences are more common in early adolescence and their significance may therefore differ from that noted in an older sample. Questionnaire outcomes may also differ from those of clinical interview. A second possible explanation for the difference in findings is sample size, with the possibility of type II error in the context of our small sample. 

### Strengths and limitations

Among the strengths of this study is the use of gold-standard clinical interviews of both parent and child to ascertain psychotic experiences and childhood trauma. Most studies on psychosis and childhood trauma have been conducted with adults, meaning there is a considerable temporal gap between events and symptom assessment, increasing the risk of recall bias. Given that our research was conducted in youths, however, the assessment of psychotic experiences took place relatively soon after the time of the traumatic events, reducing recall bias risk. A further strength of this study is that we have controlled for important potential confounders to a gene environment interaction, particularly cannabis use [69]. 

A number of limitations must be considered in evaluating the results of this study. Firstly, psychotic symptoms and trauma history were determined simultaneously, meaning that we cannot be certain that childhood trauma preceded psychotic experiences. Furthermore, the timing of trauma was not clearly ascertained. Recent findings suggest that the trauma is likely to be a causal risk factor for psychotic experiences but that this relationship is also partly bi-directional [26]. However, data on temporality is not available in this case. Secondly, the analysis was performed by pooling two samples in order to increase overall sample size and power. The study populations were broadly similar but differed in age and in the prevalence of psychotic experiences. This difference in psychotic experience prevalence was explained and adjusted for by including a variable for school grade (reflecting both study group and age) in analyses. Furthermore, the sampling methodology resulted in recruitment of a relatively small number from larger surveys, opening up a risk of selection bias. However, those who participated in the ABD study had similar psychopathology scores on the SDQ to those who did not participate, while the CT study included a representative control group without significant SDQ psychopathology. This indicates that the control group for this study (those without psychotic symptoms) differed slightly in their composition. However, this was controlled for in analysis. A further limitation of this study is the sample size. The borderline significance of the interaction between COMT genotype and childhood trauma indicates that chance should be considered a possible explanation of this interaction, though the test for interaction is conservative, making this less likely. The low study numbers limited the power of this study to detect weaker associations between genotype and psychotic experiences and between genotype interacting with non-Val-Val genotypes. However, this was not the main hypothesis of this study. Given the low study numbers, the findings presented must be regarded as preliminary and in need of replication.

## Conclusion

Our findings provide evidence in support of a gene-environment interaction between the COMT-Val158Met polymorphism and traumatic experiences in terms of risk for psychotic experiences in the population. This adds to the existing literature suggesting increased paranoia [70], schizotypal traits [41] and psychotic experiences [39,40] among Val-Val individuals in the context of stress or trauma. Specifically, young people with childhood traumatic experiences who had the COMT Val-Val genotype were more likely to experience psychotic experiences compared with Val-Met and Met-Met individuals. Clinically, this may suggest that this group could benefit from closer monitoring and support in the context of childhood trauma. Further research investigating this interaction in larger populations over a longer period would assist in clarifying the clinical prognosis and whether this population could benefit from early clinical interventions. Furthermore, such research will help to confirm whether genetic risk is specific to psychotic disorder or is, in fact, related more broadly to a broader phenotype.
